# Significant Weight Gain With the Administration of Lurasidone and Valproic Acid in Schizophrenia and Bipolar Disorder Patients

**DOI:** 10.7759/cureus.49005

**Published:** 2023-11-18

**Authors:** Shohei Okura, Yasuhiko Deguchi, Tetsuji Cho, Yuki Kageyama, Koki Inoue

**Affiliations:** 1 Neuropsychiatry, Osaka Metropolitan University Graduate School of Medicine, Osaka, JPN; 2 Psychiatry, Shigisan Hospital Heartland Shigisan, Nara, JPN

**Keywords:** schizophrenia, mood stabilizer, weight gain, valproic acid, lurasidone

## Abstract

Antipsychotics are frequently used to treat psychiatric disorders and have been associated with weight gain. Mental disorders are likely to reduce patients’ quality of life. Unhealthy lifestyles such as reduced physical activity, sleep disturbances, and irregular diets can lead to weight gain. Herein, we report two cases of schizophrenia and bipolar disorder who had a 10-kg gain in weight in six months with the administration of lurasidone and valproic acid. Lurasidone has fewer side effects, such as weight gain and somnolence. However, concomitant use of sedating antipsychotics or mood stabilizers in the acute phase and multiple doses increase the risk of weight gain. Additionally, various factors, including psychiatric symptoms and lifestyle changes, are believed to contribute to weight gain, and a comprehensive approach should be followed.

## Introduction

The prevalence of obesity, metabolic syndrome, and diabetes in patients with psychiatric disorders is found to be high, and these metabolic disorders are associated with lower life expectancy and quality of life [1]. Antipsychotics, which are often used to treat psychiatric disorders, are associated with adverse events such as weight gain, a phenomenon known as antipsychotic-induced weight gain. Lurasidone has a minimal effect on body weight because of its pharmacological profile [2]. Loebel et al. reported that the proportion of patients with schizophrenia having ≥7% increase in weight after six weeks was 2.2% in the lurasidone 20 mg/day group, 3.8% in the lurasidone 80 mg/day, and 2.8% in the placebo group [3]. To the best of our knowledge, there are only a few reports about weight gain while using lurasidone and valproic acid (VPA). In this study, we describe two cases of schizophrenia and bipolar disorder, who observed a 10-kg weight gain in six months during the administration of lurasidone and VPA, and discuss the possible mechanisms.

## Case presentation

Case 1

A 45-year-old Japanese woman with persecutory delusions and auditory hallucinations that had begun a year before was admitted to our department. When she was in junior high school, her mother was diagnosed with schizophrenia, during which she took care of her mother. Later, her mother's schizophrenia remitted, after which she started living on her own and started working as a clerk. When she was 44 years old, her mother’s schizophrenia relapsed and she started taking care of her again. Around that time, she herself was experiencing persecutory delusions and auditory hallucinations. She resigned from her job because she frequently ran into trouble with co-workers. Convinced that she could acquire a severance package from her company, she began making uncoordinated efforts to obtain one. She was taken to the hospital by her family when her disorganized behavior continued. Physical examinations revealed that she weighed 46 kg and had a body mass index (BMI) of 19.1 kg/m^2^. She had no history of any other comorbid medical illnesses. We ruled out the shared psychosis as she had no longer lived with her mother. We diagnosed her with schizophrenia and started inpatient treatment. Lurasidone was initiated at a dose of 40 mg/day. Gradually, we increased the dose of lurasidone to 80 mg/day. At the same time, we added VPA (400 mg/day) and increased the dose up to 600 mg/day, because she was poorly sedated and boisterously threatened other patients. Later, she could gradually control her behavior, and her psychiatric symptoms gradually improved. She understood the need for continuous treatment and was discharged on the 68th day of hospitalization. On discharge, no change was observed in her weight since admission. She continued her outpatient visits and demonstrated a willingness to return to society. However, she gained 10 kg in three months, weighing 56 kg with a BMI of 23.1 kg/m^2^, and complained of somnolence and akathisia; therefore, the dose of lurasidone was reduced to 60 mg/day. Thereafter, her somnolence and akathisia improved and her psychiatric symptoms did not worsen; however, her weight did not change over the next three months.

Case 2

A 38-year-old woman with a depressed mood and fatigue for the previous two weeks was admitted to our department. When she was 20 years old, she was diagnosed with depression, due to work stress. Her condition worsened when she turned 23 and had to be hospitalized. However, she continued to experience a depressive state and psychomotor agitation, necessitating frequent hospitalization. Finally, at the age of 37, she was transferred to our hospital for outpatient care for depression. We diagnosed her with the depressive phases of bipolar disorder and started inpatient treatment. We prescribed 8 mg of blonanserin, 500 mg of lithium carbonate, and 500 mg of VPA. When she was 38, her depressed mood, fatigue, and insomnia further worsened, and she was admitted to our hospital. On physical examination at admission, she weighed 80 kg and had a BMI of 32.5 kg/m^2^. Lurasidone was initiated at a dose of 20 mg/day and was increased to 40 mg/day, and lithium carbonate and blonanserin were discontinued. Later, her condition gradually improved, and she was discharged on the 28th day of hospitalization. On physical examination at discharge, she weighed 79 kg and had a BMI of 32.1 kg/m^2^. She had a history of diabetes mellitus and was receiving treatment for the same. She was prescribed metformin and her diabetes was well controlled with an HbA1c of 6.0%. She continued her outpatient visits, and her depressive mood did not relapse; however, she gained 10 kg weight in five months and weighed 89 kg, with a BMI of 36.1 kg/m^2^. We gave her exercise instructions, but her weight did not change during the next two months.

## Discussion

Here, we have reported two cases of schizophrenia and bipolar disorder in which significant weight gain was observed with the administration of lurasidone plus VPA, and explored the possible mechanisms of this observation. Because the patient in the first case resisted the weight gain and somnolence associated with antipsychotics, we chose lurasidone based on its pharmacological profile. However, as lurasidone had a poor sedative effect in the early stages of treatment, VPA was required for sedation. Although her psychiatric symptoms improved, she gained 10 kg and had a BMI increase of 4.2 kg/m^2^ in three months. In the second case, we prescribed VPA as a mood stabilizer and added lurasidone because of its weak antidepressant effect. Although her depressive mood improved, she gained 10 kg and had a BMI increase of 4.0 kg/m^2^ in five months. We consider weight gain in these two cases from three perspectives.

First, the effect of drugs, such as VPA and lurasidone, can be considered. As per previous reports, VPA often causes weight gain in 10%-70% of cases. Although the mechanism is not clear, various factors are involved, including dysregulation of the hypothalamic system, effect on adipokine levels, hyperinsulinemia, insulin resistance, and genetic susceptibility [4]. Conversely, lurasidone has a minimal effect on body weight because of its pharmacological profile. It has low affinity for histamine (H1) and serotonin (5-HT2C) receptors and has a minimal effect on weight gain. Loebel et al. reported that the proportion of patients with schizophrenia having ≥7% increase in weight after six weeks was 2.2% in the lurasidone 20 mg/day group, 3.8% in the lurasidone 80 mg/day group, and 2.8% in the placebo group [3]. A six-week study of lurasidone (dose, 20-120 mg/day) in the depressed phase of bipolar disorder demonstrated that the mean trough levels for lithium and VPA did not change significantly after six weeks of lurasidone treatment, and neither lithium nor VPA had a significant influence on the serum lurasidone area under the plasma concentration-time curve from time 0 to t after administration (AUC_0-t_) [5]. Contrastingly, a randomized controlled trial comparing lurasidone and placebo groups in patients with bipolar disorder on VPA or lithium for up to 28 weeks showed that the incidence of weight gain was higher in the lurasidone group than in the placebo group (9.8% and 5.2%, respectively; significant difference was not reported) [6]. Therefore, we consider the effect of VPA on weight gain to be high.

The second perspective is the effect of psychiatric symptoms. In persons with serious mental illnesses, such as bipolar disorder and schizophrenia, deficits in executive function and memory, residual psychotic symptoms, poor illness and self-management skills, and substance misuse can interfere with adopting and thoroughly learning new behaviors [7]. These may limit one's access to physical fitness and healthier diets. In the first case, positive symptoms such as active hallucinations and delusions improved, but the patient rarely left her home and led an autistic lifestyle. Additionally, she was more concerned about her physical appearance and adopted a diet based on healthy foods and a moderate exercise routine; however, negative symptoms such as emotional numbing, decreased motivation, and poor concentration remained. Her diet changed to the one based on processed foods and her exercise routine decreased, which is thought to have affected her weight gain. In the second case, interest in food increased, thereby causing an increase in food intake with improvement in depressive symptoms, such as poor appetite, low motivation, and decreased interest.

The final perspective is the effect of changing lifestyles, including life rhythm, work, exercise habits, nutritional balance, and sleep. Patients with bipolar disorder have a significantly higher prevalence of obesity compared with the general population, suggesting associations with lifestyle factors, such as lack of exercise, poor diet, smoking, and substance abuse [8]. Other reports indicate that negative discrimination and stigma in people with schizophrenia can hinder training, education, and close relationships, leading to social isolation [9]. These may limit access to physical fitness as well as healthier diets.

In both cases, during their hospitalization, the patients ate a nutritionally balanced diet and led an ordered life; however, after discharge from the hospital, they started preparing their own meals, which led to a breakdown in nutritional balance and eventually an irregular lifestyle. Both patients had been working before their hospitalization but left their jobs when they were hospitalized. After discharge from the hospital, they spent more time at home and their life rhythms became irregular, for example, irregular sleep schedules that included daytime sleeping. Moreover, their dietary habits also changed, with an increase in the intake of mainly processed foods and snacking between meals.

## Conclusions

Weight gain is a common adverse event observed while taking antipsychotic medication. Lurasidone is one of the drugs, which is most likely to be continued in patients with schizophrenia and bipolar disorder, as it is associated with fewer side effects such as weight gain and somnolence. However, it has a weak sedative effect and often requires concomitant use of sedating antipsychotics or mood stabilizers in the acute phase, and multiple doses increase the risk of weight gain. Additionally, various factors, including psychiatric symptoms and lifestyle changes, are believed to contribute to weight gain, and a comprehensive approach should be followed.
